# Hypomagnesemia is associated with new-onset diabetes mellitus following heart transplantation

**DOI:** 10.1186/s12933-019-0939-5

**Published:** 2019-10-11

**Authors:** Yael Peled, Eilon Ram, Jacob Lavee, Alexander Tenenbaum, Enrique Z. Fisman, Dov Freimark, Robert Klempfner, Leonid Sternik, Michael Shechter

**Affiliations:** 1 Leviev Cardiothoracic and Vascular Center, Sheba Medical Center, Tel Hashomer, Israel; 2 Sackler Faculty of Medicine, Tel Aviv University, Tel Aviv, Israel

**Keywords:** Serum magnesium, Diabetes mellitus, Heart transplantation

## Abstract

**Background:**

Diabetes mellitus (DM) is a major cause of morbidity and mortality following heart transplantation (HT), with 21% and 35% of survivors being affected within 1 and 5 years following HT, respectively. Magnesium deficiency is common among HT patients treated with calcineurin inhibitors and is a known risk factor for DM in non-HT patients. We therefore investigated the association between serum Mg (s-Mg) levels and new-onset diabetes after transplantation (NODAT).

**Methods:**

Between 2002 and 2017, 102 non-DM HT patients were assessed. In accordance with the mean value of all s-Mg levels recorded during the first year post-HT, patients were divided into high s-Mg (≥ 1.8 mg/dL) and low s-Mg (< 1.8 mg/dL) groups. The endpoint was NODAT, defined according to the diagnostic criteria of the American Diabetes Association.

**Results:**

Baseline clinical and demographic characteristics for the high (n = 45) and low s-Mg (n = 57) groups were similar. Kaplan–Meier survival analysis showed that 15-year freedom from NODAT was significantly higher among patients with high vs low s-Mg (85% vs 46% log-rank test, p < 0.001). Consistently, multivariate analysis adjusted for age, gender, immunosuppression therapies, BMI and mean creatinine values in the first year post-HT, showed that low s-Mg was independently associated with a significant > 8-fold increased risk for NODAT (95% CI 2.15–32.63, p = 0.003). Stroke rate was significantly higher in patients with low s-Mg levels vs high s-Mg (14% vs 0, p = 0.025), as well as long term mortality (HR 2.6, 95% CI 1.02–6.77, p = 0.05).

**Conclusions:**

Low s-Mg level post-HT is an independent risk factor for NODAT in HT patients. The implications of interventions, focusing on preventing or correcting low s-Mg, for the risk of NODAT and for clinical outcomes should be evaluated.

## Background

Heart transplantation (HT) is currently the “gold standard” therapy for selected patients with end-stage heart failure. Still, despite significant advances in the field, this treatment is associated with high rates of morbidity and mortality. A major cause of morbidity and mortality following HT is diabetes mellitus: it is known that at 1 and 5 years following HT, 21% and 35% of survivors, respectively, suffer from diabetes [1]. In addition to pre-existing diabetes, new-onset diabetes after transplantation (NODAT) may also develop as a complication that has a detrimental impact on patient survival or on other transplant-related adverse events [2].

Another unfavorable post-HT occurrence may be the development of a magnesium deficiency, to which a possible contributory factor is the administration of calcineurin inhibitors (CNIs), which are known to induce magnesium urinary wasting [3, 4]. Moreover, MiR-133a-regulated calcineurin-nuclear factor in activated T cells c4 (NFATc4) signalling and DNA methyltransferases-1 (DNMTs-1)-3a is changed in diabetic hearts and has been shown to be associated with a hypertrophic response and cardiac remodeling [5]. Indeed, it has been reported that hypomagnesemia frequently develops within the first few weeks following kidney transplantation, with a nadir in the serum magnesium (s-Mg) level in the second month post-transplantation and persistent hypomagnesemia is invariably accompanied by myocardial magnesium depletion in the transplanted heart [6]. Several studies have indicated that magnesium deficiency is nonetheless a potentially modifiable risk factor for diabetes in both non-transplant patients and in kidney transplant recipients [7–9]; it is also known that magnesium in the high-normality range is associated with a lower cardiovascular risk [10] and its levels are independently and inversely associated with prediabetes and overt diabetes [11]. The molecular basis for the involvement of magnesium in the pathogenesis of diabetes may lie in its role as a co-factor in several pathways, including glucose transport and insulin sensitivity and secretion [8, 12]. In healthy individuals, binding of insulin to insulin receptor in vitro leads to translocation of magnesium to platelets, leading to reduced platelet aggregation and decreased release of pro-aggregatory agents like thromboxane; this protective effect is lost in diabetics [13].

Given the high prevalence of both hypomagnesemia and diabetes in HT patients, and the proposed association of low s-Mg with an increased risk for diabetes in non-transplant patients and for NODAT in kidney transplant recipients, we designed a study to determine the association between hypomagnesemia and the incidence of NODAT in HT patients.

## Methods

### Study design and participants

A retrospective cohort study was conducted on all consecutive patients ≥ 18 years of age who underwent primary HT and follow-up at our medical center from January 2002 to August 2017. The exclusion criteria were the absence of s-Mg measurements, death within the 12 months post-transplant, pre-HT diabetes, or diabetes diagnosed within the first 12 months after HT. Data for each patient were systematically recorded upon intake and during each subsequent visit or medical contact. Donor data were obtained from the National Organ Transplantation Center and from the medical records at the hospitals at which the donors had died. Levels of s-Mg were determined using a colorimetric assay kit (Xylidyl Blue-I Method), and s-creatinine levels, by the kinetic alkaline picrate (Jaffe’s) method. Average magnesium levels during the first 12 months following HT were determined for each patient. Low s-Mg was defined as a mean s-Mg level of < 1.8 (mg/dL) [7]. The institutional protocol for post-transplant immunosuppression therapy was consistent throughout the study period and comprised a CNI, a mycophenolate-based drug, a corticosteroid, and a polyclonal induction agent. From the time that everolimus appeared on the market, it was given to a minority of patients later in the follow-up, combined with a low dose of CNI. Conversion to everolimus was dictated by the patient’s risk profile, with the considerations including cytomegalovirus infection, renal failure, allograft vasculopathy and malignancy risk. The study was approved by our institutional review board.

### Outcomes

Because mean s-Mg of all the s-Mg levels recorded during the first year after HT was the predictor, all outcome measures were assessed from 1 year after HT and beyond. The primary endpoint was NODAT, defined according to the diagnostic criteria of the American Diabetes Association, i.e., hemoglobin A1c level ≥ 6.5%, fasting plasma glucose ≥ 126 mg/dL, or random plasma glucose ≥ 200 mg/dL [14]. Secondary outcomes included: (1) all-cause mortality, (2) rejection, and (3) stroke. Stroke was defined according to the updated definition statement of stroke from the American Heart Association/American Stroke Association [15]. Rejections were diagnosed by routine or clinically indicated endomyocardial biopsy (EMB) and classified according to the revised ISHLT classification system for rejection [16]. Any treated rejection (ATR) that was clinically significant was defined as an event that led to acute augmentation of immunosuppression in conjunction with an ISHLT ≥ 2R right ventricular EMB result or non-cellular rejection (biopsy-negative rejection) with hemodynamic compromise [17]. For each patient, two rejection scores were calculated, as follows. (1) Total rejection score (TRS), as a measure of the severity of the rejection, was calculated according to the following weighting: 0R = 0, 1R = 1, 2R = 2, and 3R = 3. (2) Any rejection score (ARS), which reflected the total number of rejections, regardless of their severity, was calculated on the basis of 0R = 0, 1R = 1, 2R = 1, and 3R = 1. Each score for each particular patient was normalized by dividing it by the cumulative scores for the total number of biopsy specimens taken during the study period for that patient [18].

### Statistical analysis

In accordance with the mean value of all s-Mg levels recorded during the first year after HT, patients were divided into high s-Mg (≥ 1.8 mg/dL) and low s-Mg (< 1.8 mg/dL) groups. Data are presented as mean ± standard deviation if normally distributed, or as median and interquartile ranges (IQRs). Continuous variables were tested using the Shapiro–Wilk’s test for normal distribution. Categorical variables were expressed as frequencies and percentages. The groups were analyzed using the χ^2^ test for categorical variables and a t-test or Mann–Whitney–Wilcoxon test, as appropriate, for normal/non-normal distributed continuous variables. Analysis of 15-year NODAT was conducted using the Kaplan–Meier curves and compared by the log-rank test.

To explore the independent association of s-Mg and outcomes, a Cox proportional hazards model for 15-year NODAT was constructed. The Cox proportional hazards model for NODAT included the following covariates: recipient s-Mg (dichotomized above or below 1.8 mg/dL), age, gender, body mass index (BMI), immunosuppressive protocol, and the mean of the serum creatinine levels in the 12-months following HT. Covariate selection was based on clinical judgment. Statistical analyses were conducted using R foundation (version 3.5.1) [19].

## Results

### Study cohort

Of the original study cohort of 177 consecutive patients for whom first year s-Mg levels were available, 23 patients who died within the first year following HT, 7 patients under the age of 18, and 45 patients who were diagnosed with diabetes mellitus prior to HT or during the first year were excluded from the final analysis. Finally, 102 patients constituted the study population, median age 50 [33, 57] years, mostly men (70%) with 41% of ischemic etiology.

**Fig. 1:**
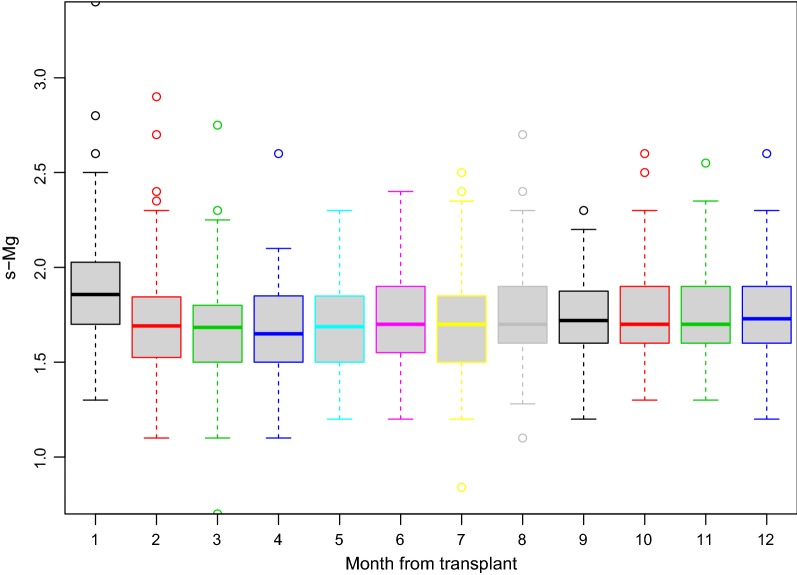
First year monthly s-Mg (mg/dL) average distribution for the total study cohort

The study cohort mean and median s-Mg levels were 1.79 ± 0.26 mg/dL and 1.73 [1.61–1.91] mg/dL, respectively. The average dispersion of s-Mg by the time (months) from HT during the first year is given in Fig. 1. In accordance with the mean value of all s-Mg levels recorded during the first year after HT, patients were divided into high s-Mg (≥ 1.8 mg/dL) (n = 45) and low s-Mg (< 1.8 mg/dL) (n = 57) groups. Baseline clinical characteristics of patients in the two groups are presented in Table 1. Baseline patient and donor clinical characteristics were similar for the two groups, except higher mean s-Mg levels in the high s-Mg group (2.0 ± 0.3 mg/dL) vs the low s-Mg group (1.6 ± 0.1 mg/dL). CNI therapies (cyclosporine vs tacrolimus) were distributed similarly in the two study groups.

**Table 1:** Baseline characteristics for the two groups

	High s-Mg Group N = 45	Low s-Mg Group N = 57	p-value
Recipient age (years) (median [IQR])	50 [37–57]	50 [31–57]	0.551
Donor age (years) (mean ± SD)	31 ± 18	27 ± 17	0.332
Recipient gender (male) (%)	32 (71)	39 (68)	0.939
Donor gender (female) (%)	40 (100)	49 (100)	1.000
Etiology (ischemic) (%)	17 (38)	25 (44)	0.677
Recipient BMI (kg/m^2^) (median [IQR])	23.7 [21.6–26.8]	23.9 [20.8–27]	0.708
Donor BMI (kg/m^2^) (mean ± SD)	24.6 ± 3.7	24.7 ± 2.7	0.886
Hypertension (%)	7 (16)	16 (28)	0.207
Dyslipidemia (%)	11 (24)	18 (32)	0.567
Past smoker (%)	9 (20)	15 (26)	0.609
Assist device (%)	8 (18)	7 (13)	0.646
Status 1 (%)	30 (67)	38 (67)	1.000
PRA > 30% (%)	0 (0)	1 (2)	1.000
Recipient blood type (%)			0.371
A	17 (42)	19 (36)	
AB	4 (10)	3 (6)	
B	5 (13)	14 (27)	
O	14 (35)	16 (31)	
Recipient creatinine (median [IQR])	1.11 [1–1.3]	1 [0.9–1.2]	0.232
Recipient bilirubin (median [IQR])	1 [0.7–1.2]	1 [0.63–1.35]	0.865
Tacrolimus^a^ (%)	19 (42)	30 (53)	0.398
Average tacrolimus^a^ (median [IQR])	13 [11.4–13.8]	12.5 [10.4–13.7]	0.400
Cyclosporine^a^ (%)	14 (31)	18 (32)	1.000
Average cyclosporine^a^ (mean ± SD)	296 ± 56	277 ± 37	0.276
Ischemic time (min) (mean ± SD)	169 ± 46	153 ± 40	0.102
PAM (mmHg) (mean ± SD)	35 ± 12	33 ± 13	0.550
CO (mean ± SD)	3.7 ± 1.2	3.5 ± 1.2	0.362
PVR (median [IQR])	2.2 [1.3–3]	2.3 [1.7–3.2]	0.468
CMV mismatch (%)	14 (39)	11 (33)	0.819
Statins post-HT (%)	41 (91)	53 (93)	1.000
Hypertension post-HT (%)	29 (64)	33 (58)	0.639
Average Mg in first year (mean ± SD)	2 ± 0.3	1.6 ± 0.1	< 0.001
Average Mg in first month (median [IQR])	2 [1.9–2.2]	1.7 [1.6–1.8]	< 0.001
Mg < 1.8 mg/dL in the first month (%)	3 (7)	38 (83)	< 0.001

*SD* standard deviation, *BMI* body mass index, *PRA* panel reactive antibody, *PAM* mean pulmonary pressure, *CO* cardiac output, *PVR* pulmonary vascular resistance, *CMV* cytomegalovirus, *HT* heart transplantation

^a^During first 3 months from HT

**Fig. 2:**
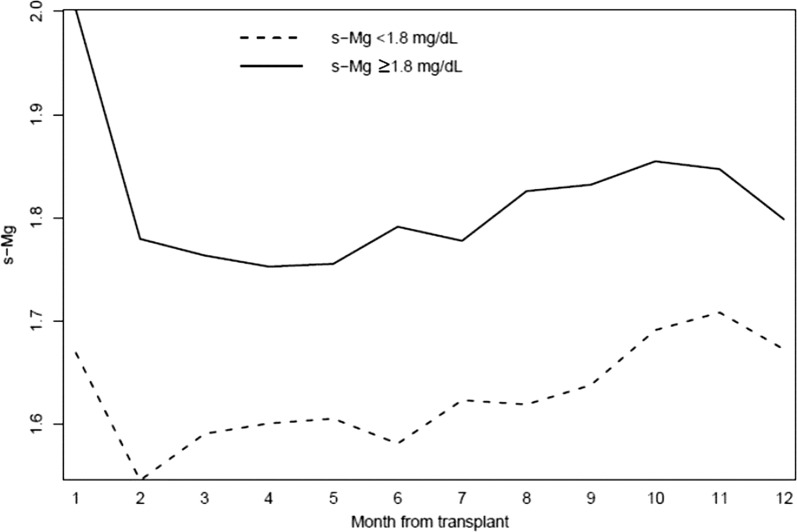
First-year monthly distribution of s-Mg (mg/dL) levels by low and high s-Mg groups

**Fig. 3:**
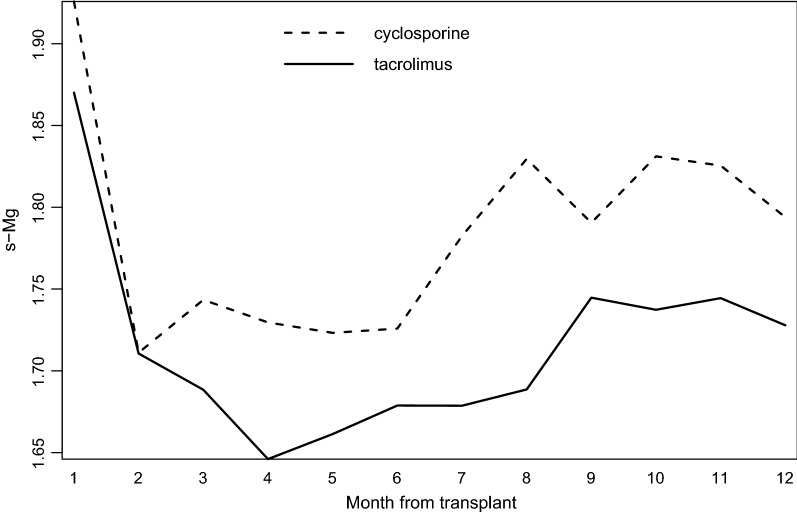
First-year monthly distribution of s-Mg (mg/dL) levels by immunosuppression therapies (patients for whom immunosuppression therapies were crossed were excluded)

Changes in s-Mg during the first year by low and high s-Mg groups are presented in Fig. 2. A s-Mg nadir was observed in the second month following HT in the low s-Mg group compared with the fourth month in the high s-Mg group, with similar courses in the two groups. Changes in monthly s-Mg during the first year by immunosuppression therapy (cyclosporine vs tacrolimus) are presented in Fig. 3. Mean s-Mg levels were lower in the tacrolimus-treated patients than in patients treated with cyclosporine, with a later s-Mg nadir (fourth month vs second month).

### Association of s-Mg level with NODAT

**Fig. 4:**
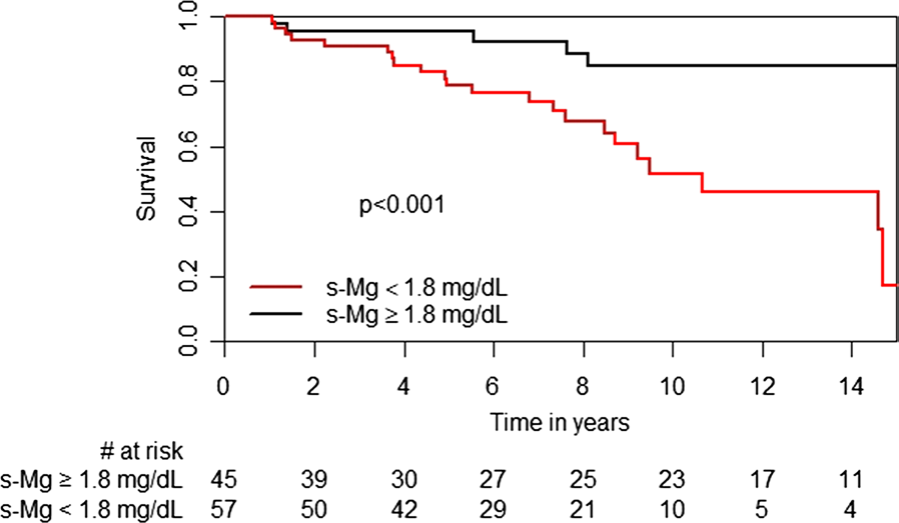
Kaplan–Meier curves for NODAT

**Fig. 5:**
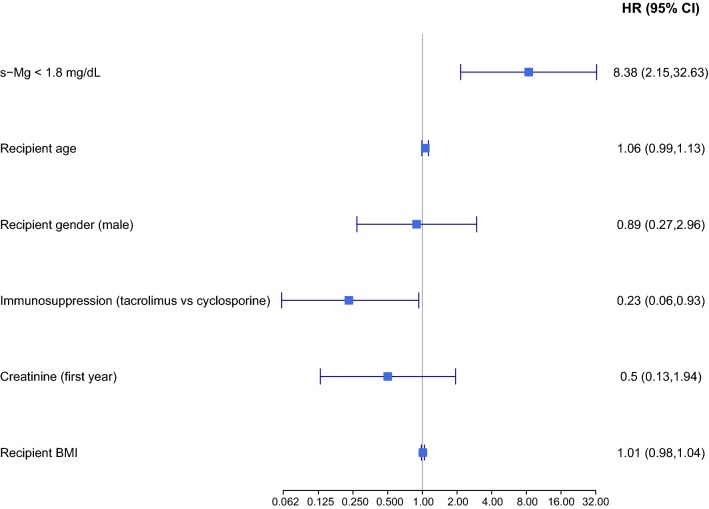
Multivariate analysis for NODAT

Kaplan–Meier survival analysis demonstrated that 15-year freedom from NODAT was significantly higher in the high s-Mg patients than in the low s-Mg patients (85% vs 46% log-rank test, p < 0.001, Fig. 4). Consistently, multivariate analysis adjusted for recipient age, gender, immunosuppression therapies, mean serum creatinine values throughout the first year post-HT, and BMI, revealed that low s-Mg was independently associated with a significant > 8-fold increased risk for NODAT (95% CI 2.15–32.6, p = 0.003, Fig. 5).

### Association of s-Mg level with other adverse clinical outcomes

**Fig. 6:**
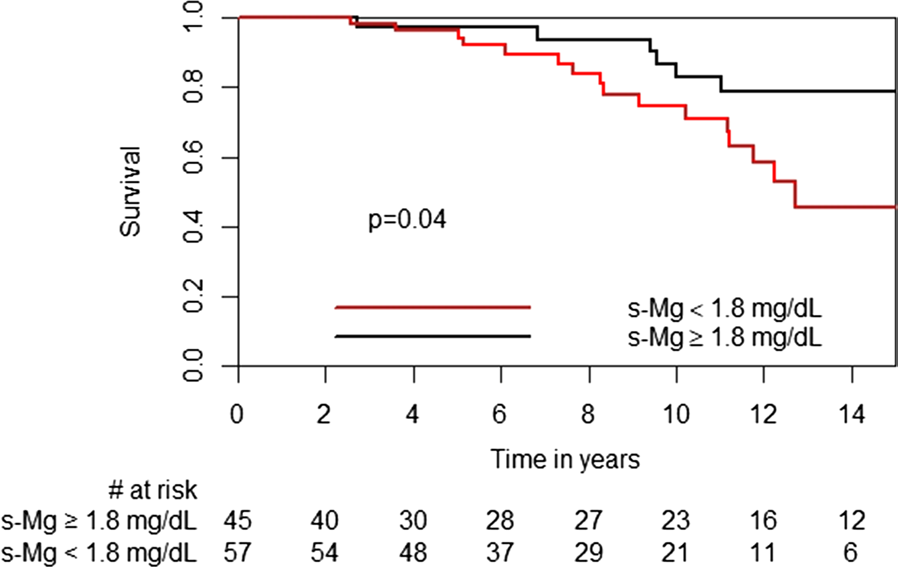
Kaplan–Meier curves for mortality

Kaplan–Meier analyses showed significantly higher mortality rates in the low s-Mg group (log-rank *p*-value = 0.04, Fig. 6). This remained consistent after adjustment for recipient age and gender (HR 2.6, 95% CI 1.02–6.77, p = 0.05). There were no differences in rejection scores between the low and high s-Mg groups (TRS (median [IQR]): 0.27 [0.12, 0.43] vs 0.33 [0.14, 0.47], *p *= 0.713; ARS (median [IQR]): 0.25 [0.12, 0.38] vs 0.29 [0.14, 0.41], *p *= 0.611, respectively), or in freedom from ATR (log-rank *p*-value = 0.289). Stroke rate was significantly higher in patients with low s-Mg levels than in those with high s-Mg (14% vs 0, p = 0.025). All strokes were ischemic, with a mean time from HT to event of 5.9 ± 4.0 years. Patients who developed stroke presented with a higher incidence of ischemic vs non-ischemic end-stage heart failure (100% vs 36%, p = 0.002), hypertension prior to HT (63% vs 19%, p = 0.018) or a history of smoking prior to HT (62% vs 20%, p = 0.023). Among these patients, more developed NODAT (75% vs 22%, p = 0.005), and the mortality rate was higher vs non-stroke patients (62.5% vs 18.1%, p = 0.013, at 15 years).

## Discussion

Diabetes is a major confounder of mortality and morbidity following HT, and therefore every effort should be made to reduce the diabetes burden in HT recipients. To the best of our knowledge, our current study is the first to demonstrate that low s-Mg after HT is independently associated with a significant > 8-fold increased risk for NODAT. Additional important findings were that the incidence of stroke was significantly higher in patients with low s-Mg levels compared to those with high s-Mg (14% vs 0, p = 0.031) as well as higher mortality rate.

Diabetes is common in HT recipients and is associated with high incidence of infection, cardiac allograft vasculopathy (CAV), graft loss, and reduced survival [20, 21]. Although risk factors for NODAT in transplant patients are similar to those in the general population (including older age, higher BMI, and male gender), immunosuppressive medications significantly further contribute to NODAT [22]. It has been shown that tacrolimus reduces insulin secretion more potently than cyclosporine both in vitro and in vivo [23, 24]. In the DIRECT trial, the incidence of NODAT was significantly lower for patients treated with cyclosporine than for those receiving tacrolimus [24]. In our study cohort, tacrolimus and cyclosporine therapies were equally distributed among the two groups, with similar mean trough levels for the two groups. This distribution, taken together with the Cox proportional hazards model, suggest that hypomagnesemia is an independent risk factor for NODAT.

Diabetes per se may also induce urinary magnesium loss, caused by hyperglycemia, hyperfiltration or a direct effect of insulin on the kidneys’ magnesium Mg channels [25]. In contrast, in the non-diabetes or pre-diabetes general population, serum glucose levels are below the threshold for urinary Mg wasting and are hence unlikely to influence s-Mg levels [8]. One of the strengths of our study is thus that by excluding patients with diabetes before the HT or those developing diabetes within the first year after HT, and following s-Mg levels with repeated measurements for 12 months post-HT, while evaluating outcomes beyond 12 months, we precluded reverse causation (as diabetes per se may induce urinary magnesium loss [25]).

Hypomagnesemia after transplantation has been attributed to a number of factors: the use of CNIs, which induce urinary loss of magnesium. In this context, a sub-population of L-type Ca2+ channels (LTCCs) has also been identified in caveolae domains that appears critical in regulating β-adrenoceptor and hypertrophic calcineurin/nuclear factor of activated T-cells (NFAT) heading to a reduced ischemic tolerance and lesser cardioprotection [26]. Additional factors are loop diuretic agents, which are frequently administered in these patients [27], and impaired gastrointestinal absorption of magnesium due to the diarrhea commonly occurring post-HT. It has been shown in kidney transplant patients and non-transplanted patients that lower s-Mg levels are an independent risk factor for new onset diabetes [7–9, 28–30]. We note that most of these studies showing an association between low s-Mg and diabetes mellitus, were based on a single measurement [8], a median or 30-day moving average assessment [9, 29], or s-Mg concentrations measured 1 year apart [8, 31]. A retrospective study of kidney transplant recipients evaluating time-dependent exposure (i.e., using 3 monthly time-varying and rolling-average s-Mg levels) indicated that low s-Mg are an independent risk factor for NODAT [7]. Another strength of our study is thus that it is based on a comprehensive assessment and repeated consistent measures of s-Mg, along with other detailed clinical parameters, allowing a detailed analysis of the study question. Our analysis thus suggests that it is the consistency of low s-Mg over time that influences the diabetes risk.

The pathophysiological mechanisms underlying the association between hypomagnesemia and NODAT are complex and have not yet been fully elucidated. It has been suggested that a number of mechanisms may provide the molecular and functional basis for the involvement of magnesium in the pathogenesis of diabetes mellitus, as follows: (1) Magnesium is a co-factor necessary for glucose metabolism in several pathways, including transport between membranes, glucose oxidation, and insulin-mediated tyrosine kinase pathways [12, 32, 33], and it may therefore be involved in insulin secretion or insulin signaling [34]. Magnesium deficiency has been shown to promote insulin resistance [32, 33, 35], and magnesium supplementation has been reported to improve both glucose tolerance and insulin sensitivity [36] in animal and clinical studies in non-transplant patients with diabetes mellitus [37, 38]. In addition, it should be pinpointed that after administration of SGLT2 inhibitors, a raise in serum magnesium concentrations (not coupled with a reduced urinary excretion) is associated with a significant improvement in endothelial function, arterial stiffness and renal resistive index [39]. (2) Common genetic variations in the magnesium-regulating genes *TRPM6*, *SLC41A2*, *CLDN19*, *CNNM2*, and *FXYD2* have been shown to significantly modify the risk of diabetes through s-Mg levels [8, 40, 41]. (3) It is also possible that mitochondrial dysfunction could underlie the association between low s-Mg and NODAT, as both hypomagnesemia and diabetes can result from mitochondrial dysfunction, as found in large pedigree with hypomagnesemia and metabolic syndrome [29, 42]. This association is further enforced by the findings in kidney transplant recipients that tacrolimus can cause secondary mitochondrial respiratory chain dysfunction [43].

Before concluding the Discussion, we touch on two findings that are relevant not only to HT patients but also to the general population at large—stroke and calcium metabolism. Prospective studies in large populations have indicated a dose-dependent inverse association between dietary magnesium and stroke incidence [44–46], with a recent meta-analysis reporting a 22% lower risk of stroke in people in the highest vs the lowest categories of dietary magnesium intake [47]. Consistent with these findings, we report here a significantly higher rate of cerebrovascular events in patients with low s-Mg. Magnesium depletion has also been associated with multiple biochemical abnormalities, among them bone and calcium metabolism. There is evidence for a suppressive effect of hypomagnesemia on parathyroid hormone secretion and resistance [48], further supporting the importance and potential clinical implications of managing low-Mg state in HT patients known to suffer frequently from osteoporosis, partially due to prolonged steroid treatment.

Our findings may have potentially important implications for the management of patients following HT. As hypomagnesemia is a potentially modifiable risk factor for diabetes and NODAT, a number of studies were conducted aiming to verify its clinical implications. In non-transplant populations higher consumption of magnesium was associated with a lower risk of diabetes [37, 38, 49, 50], and in a randomized trial of kidney transplant patients, magnesium supplementation 3 months post-transplant, was shown to improve fasting blood glucose levels [51]. The increased s-Mg level observed following SGLT2 inhibition, combined with a favourable cardiovascular profile of these drugs, are suggestive of a therapeutic potential for diabetic and pre-diabetic HT recipients, that should be further studied.

There are several limitations to our study. First, there is the limitation inherent in observational trials that uncovers associations but precludes the determination of cause-and-effect relationships. Second, this study was limited by being based on a single-center experience. Third, measuring s-Mg and not intracellular magnesium levels may influence the assessment of the patients’ magnesium status. Finally, magnesium intake was not controlled or assessed. Nevertheless, the intake of magnesium should be reflected in the measured s-Mg. Thus, any conclusions drawn from the data must be replicated with a larger sample size and a prospective study design.

## Conclusion

In conclusion, our study demonstrates that low post-HT s-Mg level is independently associated with an increased risk for NODAT in HT patients. Nevertheless, the implications of interventions, focusing on preventing or correcting low s-Mg, on the risk for NODAT and on clinical outcomes should be further evaluated.

## Data Availability

Data collected from a departmental database.

## Abbreviations

ARS: any rejection score
BMI: body mass index
CAV: cardiac allograft vasculopathy
DM: diabetes mellitus
EMB: endomyocardial biopsy
HT: heart transplantation
ISHLT: International Society of Heart and Lung Transplantation
NODAT: new-onset diabetes after transplantation
TRS: total rejection score
